# Describing interaction effect between lagged rainfalls on malaria: an epidemiological study in south–west China

**DOI:** 10.1186/s12936-017-1706-2

**Published:** 2017-01-31

**Authors:** Yunyun Wu, Zhijiao Qiao, Nan Wang, Hongjie Yu, Zijian Feng, Xiaosong Li, Xing Zhao

**Affiliations:** 1 West China School of Public Health, Sichuan University, No. 17 Section 3, South Renmin Road, 610041 Chengdu, China; 2 Division of Infectious Disease, Key Laboratory of Surveillance and Early-Warning on Infectious Disease, Chinese Center for Disease Control and Prevention, Beijing, China; 3 Office for Disease Control and Emergency Response, Chinese Centre for Disease Control and Prevention, NE Pacific Street, 102206 Beijing, China

**Keywords:** Malaria, Rainfall, Lag, Nonlinear, Interaction

## Abstract

**Background:**

When discussing the relationship between meteorological factors and malaria, previous studies mainly focus on the interaction between different climatic factors, while the possible interaction within one particular climatic predictor at different lag periods has been largely neglected. In this study, this issue was investigated by exploring the interaction of lagged rainfalls and its impact on malaria epidemics, which is a typical example of those meteorological variables.

**Methods:**

The weekly data of malaria cases and three climatic variables of 30 counties in southwest China from 2004 to 2009 were analysed with the varying coefficient-distributed lag non-linear model. The correlation patterns of the 6th, 9th and 12th week lags would vary over different rainfall levels at the 4th-week lag.

**Results:**

The non-linear patterns for rainfall at different rainfall levels are distinct from each other. In the low rainfall level at the 4th week lag, the increasing rainfall may promote the transmission of malaria. However, for the high rainfall level at the 4th week lag, evidence shows that the excessive rainfall decreases the risk of malaria.

**Conclusion:**

This study reports for the first time that the interaction effect between lagged rainfalls on malaria exists, and highlights the importance of integrating the interaction between lagged predictors in relevant studies, which could help to better understand and predict malaria transmission.

**Electronic supplementary material:**

The online version of this article (doi:10.1186/s12936-017-1706-2) contains supplementary material, which is available to authorized users.

## Background

Malaria is one of the most severe mosquito-borne infectious diseases that threaten human health around the globe [1]. Since the primary vector for malaria is *Anopheles*, the dynamics and distribution of malaria are closely correlated with meteorological conditions [2]. From a biological perspective, climatic factors have a profound effect on malaria incidence as they affect both the development of mosquitoes and malaria parasites within mosquitoes [3, 4]. For instance, plentiful rainfall and increased humidity offer mosquitoes suitable sites to breed, resulting in increasing number of mosquitoes. Moreover, appropriate temperatures promote malaria prevalence by enhancing the mosquito’s growth and biting rate, as well as increasing the viability and development rate of parasites within vectors [5].

When exploring the meteorological effect on malaria incidence, special attention should be paid to two particular issues, which are the lag and non-linear patterns [6, 7]. In some previous studies, lag time was assumed to be single fixed [8–11]. This is unreasonable, especially when describing the relationship between climatic variables and malaria risk at the large population level. Biologically speaking, there are at least two stages that should be considered for the lag effect, such as the development of mosquitoes and the incubation of parasites within the mosquito. The lag time of each stage may vary indefinitely based on climatic conditions, thus generating a smoothly varied lag distribution at population level. The non-linear correlation between rainfall and malaria incidence has been acknowledged and validated both experimentally and epidemiologically in a series of existing studies [12, 13]. It is proposed that similar non-linear effect may also exist for temperature [14–16]. Therefore, both lag and non-linear patterns should be considered in the model. For this purpose, the distributed lag non-linear model proves a valuable and effective method [17].

When discussing the relationship between meteorological factors and malaria incidence, however, in most previous epidemiological studies, the effect of between-lag interaction has long been overlooked. Unlike the interaction between different exposure variables where the various climatic factors are of the same time-period and simultaneously affect malaria incidence, the between-lag interaction is defined as the interaction between one covariate at different lag time, such as the interaction effect of the rainfalls four and five weeks previously on malaria risk for the current week. The concept of lagged interaction was first developed by Heaton in 2014, as he reported the corresponding statistical methods to examine the relationship between heat exposure and mortality [18]. When considering the between-lag interaction, the total effect of the climatic variables is not simply an accumulation of lagged effects, as is conventionally supposed. This is explained by the fact that the number of mosquitoes at week *t* are substantially dependent on the rainfall at week *t* *−* *1*. More specifically, adequate rainfall at week *t* *−* *1* provides mosquitoes with abundant breeding sites and promotes their development, which leads to an increased number of mosquitoes at week *t*, and consequently a greater risk of malaria transmission.

This study aims to investigate the interaction effect between climatic factors at different lag periods on malaria risk, exemplified by rainfall. So far, it has not been directly reported in previous studies. Specifically, with weekly data during the period of 2004–2009 among 30 counties in southwest China, a varying coefficient distributed lag non-linear model was applied to investigate the association between rainfall and malaria cases. The correlation pattern between rainfall and malaria incidence was set to change depending on the three levels of rainfall at the fourth week lag. The results can help researchers to better understand the complex relationship between climatic factors and malaria transmission, and develop potential better prediction models.

## Methods

### Study sites

The southwestern region of China has been severely threatened by malaria in the last century [19]. After decades of continuous effort, malaria in these provinces has been brought under control. However, due to the effect of global warming, as well as the complex meteorological condition in this region (most counties are of sub-tropical climate; a few southern counties in the tropical region) [20], malaria poses a potential threat to the health of its populace.

The southwest region of China encompasses a large area from 21°14′N to 34°31′N and 97°35′E to 110°19′E. It covers primarily four provinces of Sichuan, Chongqing, Yunnan, and Guizhou with 483 counties in total (county-level cities and districts). This region has a population of 189,977,077 (results from the sixth national census in 2010) and occupies an area of 1,137,570 sq km. Malaria data covered 483 counties, while only 131 counties had daily meteorological records. Chosen from those counties where malaria and climatic data were available, 30 counties with the highest average annual incidence were chosen for this study. The relevant principles of sample selection have been described in a previous study [21]. Additional file 1 shows the 483 counties and the selected 30 counties in southwest China.

### Data description

The weekly meteorological data from July 2003 to December 2009 was collected from the Chinese Meteorological Data Sharing Service System [22], in which the mean temperature (°C), rainfall (mm) and relative humidity (%) had been recorded. There are 131 meteorological monitoring stations among the 483 counties in the southwest region, and those that are relevant to the counties with high malaria incidence were used.

As daily malaria records would bring many zero counts and jeopardize the stability of the parameter estimation, weekly case reports were used in this study and collected through the Chinese Information System for Infectious Diseases Control and Prevention (CISIDCP) from 2004 to 2009 among the 30 counties in southwest China [23]. Although both malaria sub-types (*Plasmodium vivax* and *Plasmodium falciparum*) could be the potential cause of the cases reported, most records did not include the type of parasites. The population data associated with the selected counties were obtained from the National Bureau of Statistics of China, from 2004 to 2009.

### Basic distributed lag non-linear model (DLNM)

The methodology of distributed lag non-linear model **(**DLNM) is used to describe the dependencies that are both non-linear and delayed [17]. Because malaria cases are ordinary count data, the association between the expected number of malaria cases *E*(*Y*_*it*_) at week *t* in county *i* and climatic variables in the previous weeks was modelled by the Poisson regression,

1 $$\begin{aligned} \log (E(Y_{it} )) &= \log (d_{it} ) + \beta_{i0}\\ & \quad + \sum\limits_{l = 4}^{15} {f(x_{i(t - l),r} ,\beta_{rl} )} \\ & \quad + \sum\limits_{l = 4}^{15} {f(x_{i(t - l),h} ,\beta_{hl} )} \\ & \quad + \sum\limits_{l = 3}^{10} {f(x_{{i(t - l),T_{m} }} ,\beta_{{T_{m} l}} )} ,\\ \end{aligned}$$

where *d*_*it*_ denotes the population in county *i* at week *t*; *β*_*i*0_ denotes the intercept effect for county *i*. *x*_*it*,*r*_, *x*_*it*,*h*_ and $\, x_{{it,T_{m} }}$ are the weekly meteorological variables for county *i* at week *t*, representing the rainfall, relative humidity and mean temperature, respectively.

The lag ranges for meteorological factors were determined based on relevant biological knowledge. Considering the lag effect, it would be logical to assume that cases in a specific week will be affected by climatic factors several weeks before. Consequently, Model (1) could be used to estimate the cumulative contributions across the entire lag range, rather than on a single fixed lag time. The lag ranges were determined by referring to those biological factors and empirical results from previous studies [24]. The period from the fourth to the 15th week was used as the lag range for weekly rainfall and mean relative humidity, while for weekly mean temperature it was from the third to the tenth week [24, 25].

There are two basis functions included in Model (1) to represent the non-linear and lag effects. Taking rainfall as an example, the first is *f*(*x*_*i*(*t* − *l*),*r*_, *β*_*rl*_), which describes the non-linear effect of rainfall that happened *l* weeks before. It could be interpreted by many functional forms, for instance, the polynomial function. As for the other function, its purpose is to constrain the parameter *β*_*rl*_, thus to refrain from the high collinearity caused by the significant correlation between rainfall on consecutive weeks. With the introduction of the constraining function, a reduction of the noise in the unconstrained distributed lag model could be achieved with less bias [7]. Subsequently, the second-order natural cubic spline was applied for the investigation of both basis functions in Model (1) due to the fact that the meteorological variables are unimodal [26], as well as the requirement for parsimony.

Correlations between climatic factors and malaria incidences are not equal from place to place, and that the correlation in one county would be comparatively larger than that within two counties, in most circumstances. The inequality was caused by some unmeasured (or even unmeasurable) county-specific variables. To deal with the potential confounding, *β*_*i*0_ is modelled as a multilevel random intercept, obeying a normal distribution that *β*_*i*0_ ∼ N(*β*_0_, *δ*_0_^2^). *β*_0_ is the average intercept of all counties, while *δ*_0_^2^ represents the variation of county-specific intercepts around *β*_0_.

### Varying coefficient distributed lag non-linear model

The lagged interaction was excluded in Model (1) due to the hypothesis that the rainfall at week *t* has the same effect at different levels of rainfall at week *t* − *k*. However, the effect of rainfall at week *t* may also depend on the level of rainfall at week *t* − *k*. In this regard, a varying coefficient model [27] was used to examine the dependencies of the rainfall effect at week *t* on the rainfall level at week *t* − *k* and to investigate the between-lag interaction.

The rainfall at the fourth week lag was set as the stratification variable in this study, mainly considering the lag range. As *x*_*i*(*t* − 4),*r*_ denotes the rainfall at the fourth week lag, *x*_*i*(*t*-4),*r*_ should, in a certain degree, influence the lag non-linear pattern of rainfall at other lag weeks, providing the hypothesis is correct that the lagged interaction exists and the effect of rainfall at other lag weeks is indeed dependent on the rainfall level at the fourth week lag.

To investigate the possible influence of the lagged interaction, all *x*_*i*(*t* − 4),*r*_ were divided into three quantile groups (33.3 and 66.6% percentiles). The three groups were denoted as *R*_*i*(*t* − 4),*r*0_, *R*_*i*(*t* − 4),*r*1_ and *R*_*i*(*t* − 4),*r*2_, which represents the *x*_*i*(*t* − 4),*r*_ at the low, medium and high level of rainfall at the fourth week lag, respectively. Model (1) is then adjusted to embody these changes, and the modifications are shown below:

2 $$\begin{aligned} \log (E(Y_{it} )) &= \log (d_{it} ) + \beta_{i0} + \sum\limits_{g = 1}^{2} {\alpha_{g} \times R_{i(t - 4),rg} } \\ &\quad + \sum\limits_{l = 4}^{15} {f(x_{i(t - l),r} ,\beta_{rl} (R_{i(t - 4),rg} ))} \\ &\quad + \sum\limits_{l = 4}^{15} {f(x_{i(t - l),h} ,\beta_{hl} )}\\ &\quad + \sum\limits_{l = 3}^{10} {f(x_{{i(t - l),T_{m} }} ,\beta_{{T_{m} l}} )} ,\\ \end{aligned}$$

Compared to Model (1), Model (2) is different in two major aspects. The first is that *β*_*rl*_ has now become *β*_*rl*_(*R*_*i*(*t* − 4),*rg*_), reflecting the fact that the coefficient *β*_*rl*_ is now varying over *R*_*i*(*t*-4),*rg*_. Consequently, the effect of rainfall at other lag weeks is now dependent on the relevant rainfall level at the fourth week lag. Besides the lag non-linear pattern, the modified model can also be used to interrogate the lagged interaction that the rainfall effect of other lag weeks changes over different levels of rainfall at the fourth week lag. If the lagged interaction indeed exists, the lag non-linear patterns for rainfall at the three levels should differ from each other.

The second difference is that the coefficient *R*_*i*(*t* − 4),*rg*_ is now included in the intercept. Like the ordinary categorical predictor, *R*_*i*(*t* − 4),*r*0_ at the lowest level was used as the reference group, and *α*_1_ and *α*_2_ represent the differential effects for *R*_*i*(*t* − 4),*r*1_ and *R*_*i*(*t* − 4),*r*2_, respectively. An assumption was made in Model (2) that the mean temperature and relative humidity do not interact with the rainfall. The reference values of all climatic factors are all set at zero.

The analysis may be sensitive to the choice of lag ranges, and therefore the situation where the fourth to 14th weeks were selected as lag range for rainfall instead of the fourth to 15th weeks was also investigated, and the results were robust and showed no significant change. All the analysis was performed with R, which is an open statistical software [28]. Specifically, the package lme4 [29] was loaded to estimate the parameters. The data above have been used in previous studies by the same research group to explore other relationships between meteorological factors and malaria incidences [21, 30].

## Results

### Descriptive analysis

Among the selected 30 counties in southwest China, 21,944 malaria cases were reported in total over the 6 years from 2004 to 2009, and the descriptive analysis of the collected data could be found in Additional file 2.

**Fig. 1:**
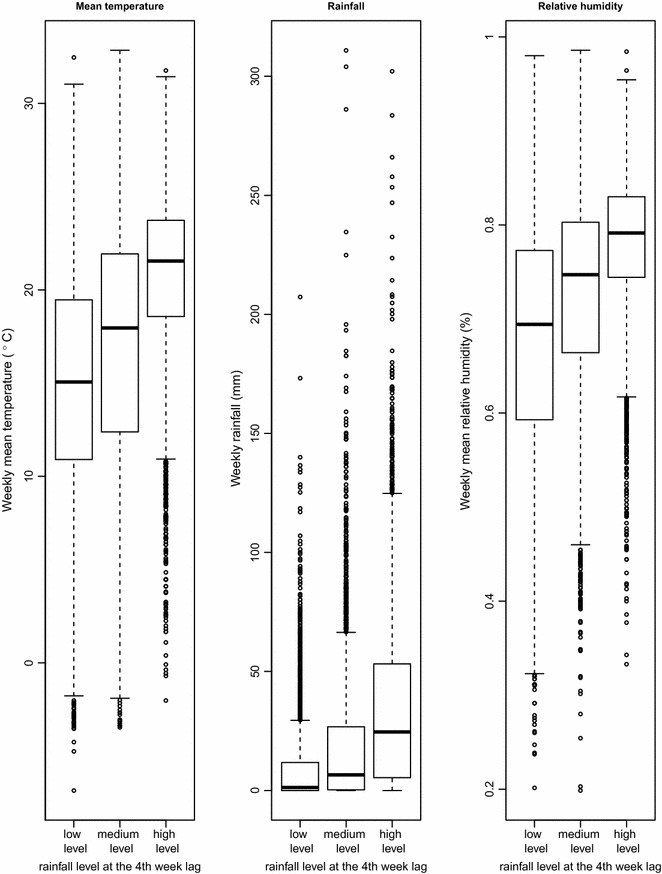
Box plot comparison of meteorological variables between three rainfall levels at the fourth week lag. The *dark line* in the *middle* of the boxes is the median value; the *bottom* and *top* of the boxes indicates the 25th and 75th percentile, respectively; *whiskers* represent 1.5 times the height of the box; *dots with numbers* represent the value of outlier cases

The three rainfall ranges at the fourth week lag are (0.0, 1.6 mm), (1.6, 20.0 mm), (20.0, 310.9 mm), and their sample sizes are 3155, 3106 and 3129, respectively. The slight difference between the sample sizes is ascribed to the fact that most weekly rainfall values are concentrated at the 33.3 and 66.6 percentile cut-points. Nonetheless, these values are eventually grouped into the corresponsive levels and cause the inequality of sample numbers. In Fig. 1, the meteorological variables between different rainfall levels have been compared, and pronounced trends were found: the mean temperature, rainfall and relative humidity were all positively correlated with the rainfall levels at the fourth week lag. Conclusively, the annualized average incidences are 9.73, 14.18 and 13.36 per 100,000 for low, medium and high rainfall levels, respectively.

### Varying coefficient distributed lag non-linear model

**Fig. 2:**
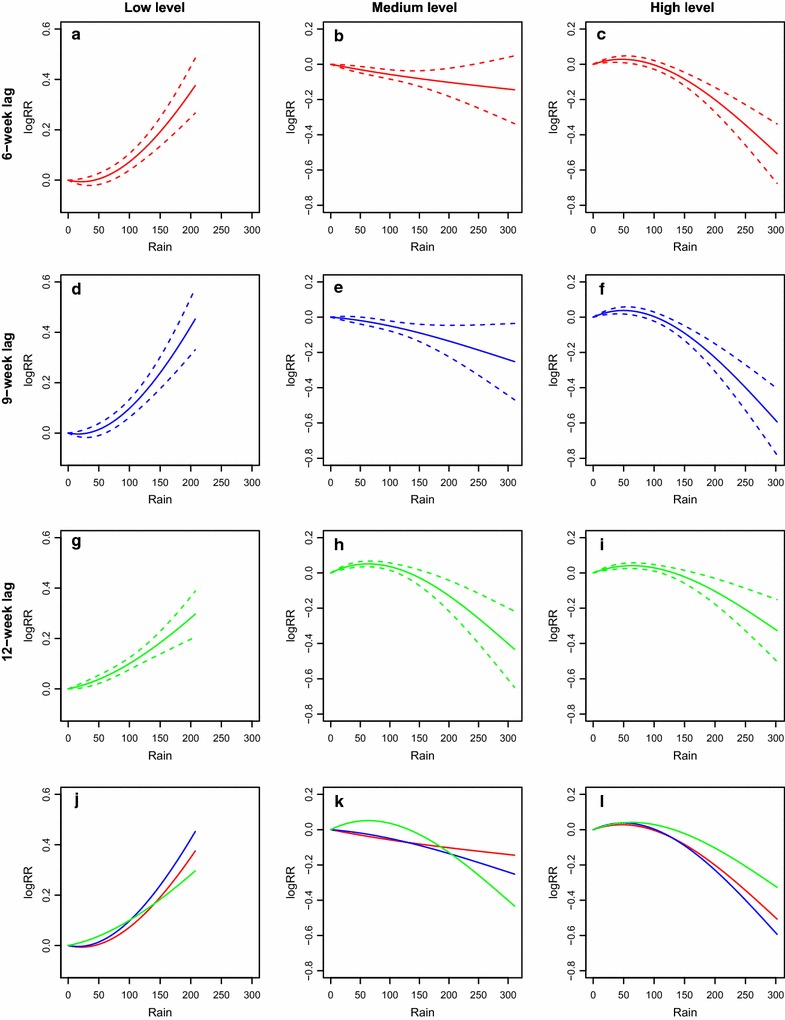
The estimation of non-linear patterns between rainfall and malaria incidences in the exposure dimension. The *Y*-*axis* represents the logarithm value of the relative risk ratio compared to the reference rainfall 0.0 mm. The *solid line* is the estimated non-linear curve, with *dashed lines* indicating its 95% confidence interval. On the one hand, the *solid lines* in the top 3 *rows* shows the scenarios for the 6th week lag (*red line*, **a**–**c**), the 9th week lag (*blue line*, **d**–**f**) and the 12th week lag (*green line*, **g**–**i**), while the *fourth row* shows the difference among the results at the 6th, 9th and 12th week lags (**j**–**l**). The first three panels in each *column* represent the specific rainfall level at the fourth week lag. Specifically, the columns of (**a**, **d**, **g**, **j**), (**b**, **e**, **h**, **k**) and (**c**, **f**, **i**, **l**) are for the low, medium and the high rainfall levels at the fourth week lag, respectively. The range of *X*-*axis* depends on the corresponding observed range of rainfall

Figure 2 demonstrates the estimated lagged non-linear relationships between rainfall and malaria incidence in the exposure dimension. The results are presented with nine representative panels, as there are three lag conditions and each condition was analysed under three levels of rainfall at the fourth week lag. The Y-axis represents the logarithm value of the relative risk ratio in comparison with the reference value at rainfall 0 mm. In general, the rainfall levels at the fourth week lag affect the non-linear patterns between rainfall and malaria incidence under each lag time. In the low rainfall level at the fourth week lag, rainfall promotes malaria incidence. On the contrary, in the high rainfall level at the fourth week lag, the risk of malaria incidence would decrease as the rainfall increases. (See figure on previous page).

For each lag time, the non-linear patterns between rainfall and malaria incidence are distinctively different across the three rainfall levels. In the high rainfall level at the fourth week lag, the magnitude and direction of the association would change according to the rainfall values. More specifically, in the high level, the logarithmic value of relative risk (logRR) increases slightly at first and reaches the maximum at approximate 75 mm, but then it starts to decline sharply. In the range 0–150 mm, rainfall is positively associated with malaria incidence. Afterwards, the correlation between rainfall and malaria risk becomes negative. The same trend is observed for the 12th week lag in the medium level (panel h of Fig. 2). Secondly, in the medium rainfall level at the fourth week lag (except panel h), the curve shows a slowly downward trend. However, this correlation is not statistically significant, as the value of 0 is included in the 95% confidence interval of logRR.

In the low rainfall level at the fourth week lag, rainfall is positively associated with malaria incidence, and the logRR would increase sharply when the rainfall increases. The ninth week lag has the greatest impact on malaria risk at the low and high rainfall levels. The effect of the sixth week lag ranks second, which is greater than that of the 12th week lag.

**Fig. 3:**
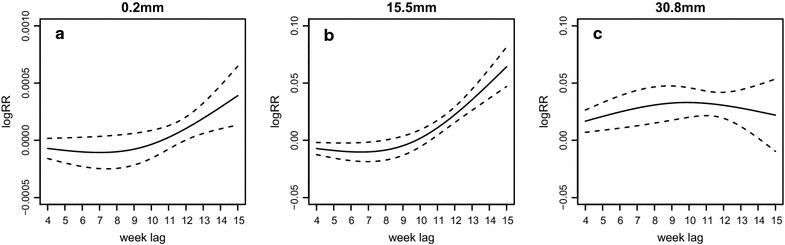
The estimation of non-linear patterns between rainfall and malaria incidence in the lag dimension. The *Y*-*axis* represents the logarithm value of the relative risk ratio compared to the reference rainfall 0.0 mm. The *solid line* is the estimated non-linear curve, with *dashed lines* indicating its 95% confidence interval. The *three panels* of **a**–**c** show the scenarios for the rainfall at 0.2, 15.5, 30.8 mm, respectively

Figure 3 shows the estimated lagged non-linear relationships between rainfall and malaria incidence in the lag dimension. The results are presented with three rainfall values at the 25, 50 and 75% percentiles of weekly rainfall from 2004 to 2009, which are 0.2, 15.5 and 30.8 mm, respectively. As the three representative rainfall values were within the three rainfall level ranges (0.0, 1.6 mm), (1.6, 20.0 mm) and (20.0, 310.9 mm), respectively, Fig. 3 also demonstrates the overall relationship between rainfall and malaria incidence for each rainfall level at the fourth week lag. Specifically, Fig. 3a shows the correlation at the low rainfall level, Fig. 3b the medium rainfall level and Fig. 3c the high rainfall level. Y-axis represents the logarithm value of the relative risk ratio in comparison with the reference value at rainfall 0 mm. The three panels present distinct non-linear patterns between rainfall and malaria incidence at different rainfall values. When the rainfall is at 30.8 mm, the corresponding distributed lag curve shows an apparent inversed U-shape, which goes up first until peaking at the ninth week, then starts going downwards and ends up with non-significant correlation from the 13th week. When the rainfall is at 15.5 and 0.2 mm, both curves show similar trends: the correlations are non-significant at first and then start to increase substantially during the 11–15th weeks. Finally, by comparing the three panels, it is observed that the logRR increases along with the rainfall values, and the increasing rate is much larger at the lower rainfall. Specifically, the increasing rate at 15.5 mm is nearly 100 times of that at 0.2 mm, while the difference becomes minimal when comparing that under 15.5 and 30.8 mm.

## Discussion

The interaction between meteorological factors is important in malaria transmission, as they are closely associated with vector abundance and survival as well as parasite maturation [24]. Exploring the interaction between climatic variables on malaria incidence can help to better understand the relationship between meteorological factors and malaria incidence [31]. The interaction between exposure predictors is common in existing studies, but so far there is no report dedicated to the lagged interaction between climatic factors in the process of malaria transmission, which may also play a crucial role in malaria epidemics. Particularly, the lagged interaction effect on malaria incidence examined in this study was the interaction between rainfall at the 4th week lag and that at the 6th, 9th and 12th week lags.

The results indicate that the rainfall at the 4th week lag affects the correlation between malaria incidence and rainfall at the other lag weeks, implying the interaction effect between lagged rainfalls on malaria. When in the low rainfall level at the fourth week lag, the malaria risk increases along with the increase of rainfall, suggesting that the increasing rainfall promotes malaria transmission when rainfall is low at the 4th week lag. In contrast, excessive rainfall decreases the risk of malaria when rainfall is high at the 4th week lag, which can be observed in panel c, f, i of Fig. 2. These results can be explained by malaria dynamics [19]: rainfall elevates the environmental humidity and brings about many temporary puddles, simultaneously increasing the number of mosquito breeding sites and enhancing mosquito survival. However, excessive rainfall and accumulation of surface water in complicated terrain would potentially destroy mosquito-breeding sites, thus reducing the mosquito density. Abundant rainfall may also prevent people from working outdoors, resulting in lower chances of people being bitten by mosquitoes and consequently, decreasing malaria incidence. Specifically, when the rainfall level at the 4th week lag is low, the greater rainfall at week *t* would relieve the effect of insufficient rainfall so that rainfall offers more breeding habitats to mosquitoes and increases their number, resulting in the increased risk of malaria incidence. In contrast, when the rainfall level at the fourth week lag is high, abundant rainfall at week *t* would exacerbate the effect of excessive rainfall, resulting in mosquito breeding sites being destroyed and people reducing their outdoor activities. The effect of high rainfall at week *t* would be attenuated or even become negative.

It is also observed that the lagged effect of rainfall on malaria incidence was greatest at the ninth lag week, compared to the 6th and 12th weeks. This is biologically acceptable as the effect of rainfall occurring in the current week or too long before is negligible on malaria incidence.

Greater rainfall brings a higher relative risk but a shorter lag for malaria cases, which can be observed from Fig. 3. Specifically, in the low and medium levels of rainfall at the 4th week lag, rainfall starts to be significantly associated with malaria incidence at the 11th week. In high rainfall level at the fourth week lag, the distributed lag curve shows a significant correlation from the 4th to 13th week. Compared with the high level of rainfall at the fourth week lag, rainfall in low levels is associated with delayed malaria risk. The results are consistent with a previous study [32]. These may be a result from the previously mentioned malaria dynamics that rainfall could provide fitted habitats for mosquitoes to breed, thus shortening their life cycles and accelerating the spreading of malaria [33]. Despite the fact that the relative risk for malaria cases is positively correlated with rainfall, the increase in the relative risk is more drastic when rainfall is low, while it becomes minimal when rainfall is high. This phenomenon may be explained by the saturation effect, where the contribution of increasing rainfall to the development of mosquito and parasite becomes negligible or even counterproductive.

Rainfall is selected as an example to reveal the lagged interaction on malaria incidence mainly for biological and epidemiological considerations. From an entomological perspective, rainfall affects most of the stages of the mosquito’s life cycle. For example, plentiful rainfall provides mosquitoes with aquatic breeding sites for their growth and reproduction [34]. While from an epidemiological view, the reported relationship between rainfall and malaria vary in the literature [24]. This is especially true in China, as several studies showed that rainfall was closely correlated with malaria incidence [16, 35, 36], while other studies denied the existence of such correlations [37]. Understanding the interaction effect between rainfall at different lag time on malaria incidence may help to better explore the relationship between rainfall and malaria incidence.

*α*_1_ and *α*_2_ are introduced to describe the main effects of the rainfall levels at the fourth week lag. As demonstrated in Fig. 1, the three groups of different rainfall levels at the fourth week lag do not have identical baseline distribution of climatic factors. Even under the same rainfall condition at the fourth week lag, the average effect of rainfall among the three groups should be distinctively different; *α*_1_ and *α*_2_ are consequently added as the average deviation, ensuring that the logRR values of all groups would be zero at the reference rainfall, and allowing the comparison of variations for rainfall.

The county-specific random intercept model allows fitting a regression model to meteorological factors with the systematic unexplained variation among the 30 counties. As with other epidemiological literature on the relationship between meteorological variables and malaria incidence, the final results would be potentially interfered by some confounding factors. For instance, there may be different preventive measures with different enforcement strength that are deployed by an individual county to fight malaria, as well as some behavioural patterns, such as the utilization of nets of different types. The variance *δ*_0_^2^ of the county-specific random intercept *β*_*i*0_ represents the variation between counties that are not caused by the climatic predictor. The random intercept model has proven efficient in handling the potential bias [38].

There are a few limitations that should be acknowledged. First, the data quality may change over the 6 years. This primarily varied with time, and the best data quality was found in 2009. Second, only 30 counties with malaria prevalence were used in this study. However, this should not introduce significant inherent bias into this study. The range of malaria incidence varied from low to high values. Specifically, the annualized average incidence ranged from 348.2/100,000 to 1.1/100,000. In particular, it is evident (see Additional file 2) that the 30 counties included many low-incidence counties, such as Eshan with just 11 malaria cases in 6 years. Therefore, the selection method in this study should not undermine the credibility of this study. Third, like several existing studies [39, 40], the characteristics of *P. vivax* and *P. falciparum* were not analysed separately due to the lack of associated information in this study. As a result, vivax relapses may be mistaken as new infections caused by meteorological factors. The lag non-linear patterns of the two malaria sub-types may be slightly different from each other. By investigating the potential bias, future studies might provide more details to elucidate the association between climatic factors and malaria incidence in southwest China.

## Conclusions

Using weekly data of malaria cases and climatic variables during the period of 2004–2009 among 30 counties in southwest China, the interaction effect between rainfall at different lag time on malaria incidence was examined. As previous studies rarely accounted for the interaction between lagged climatic factors, this work highlights the importance of including the lagged interaction effect in the investigation of malaria incidence, which can provide supplementary evidence to understand and predict malaria transmission.

## Additional files

**Additional file 1.** The map of the 483 counties in southwest China and the selected 30 counties [21]. The grey-coloured counties are the 30 top incidence counties with both malaria and meteorological data.

**Additional file 2.** Characteristics of the 30 studied counties [31].

## Abbreviations

DLNM: distributed lag non-linear model
logRR: logarithmic value of relative risk
