# Supplementary figures and images for: Describing interaction effect between lagged rainfalls on malaria: an epidemiological study in south–west China
Source: Malar J. 2017 Jan 31;16:53. doi: 10.1186/s12936-017-1706-2 (PMC5282846; doi:10.1186/s12936-017-1706-2)

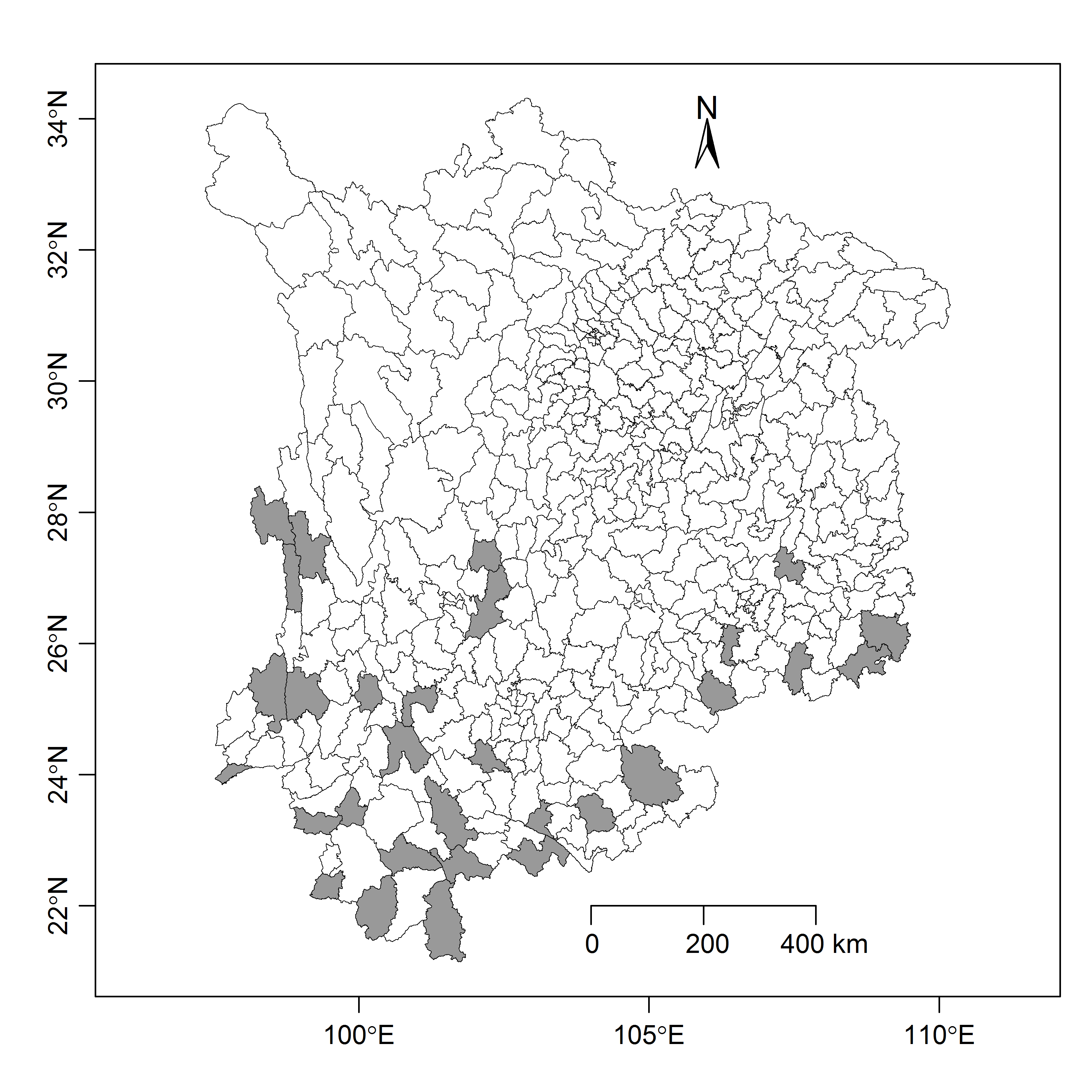

Supplement: Supplementary file 1 — Additional file 1. The map of the 483 counties in southwest China and the selected 30 counties [21]. The grey-coloured counties are the 30 top incidence counties with both malaria and meteorological data. [file 12936_2017_1706_MOESM1_ESM.png]
