# Supplementary material for: Describing interaction effect between lagged rainfalls on malaria: an epidemiological study in south–west China
Source: Malar J. 2017 Jan 31;16:53. doi: 10.1186/s12936-017-1706-2 (PMC5282846; doi:10.1186/s12936-017-1706-2)
Supplement: Supplementary file 2 — Additional file 2. Characteristics of the 30 studied counties [31]. [file 12936_2017_1706_MOESM2_ESM.docx]

**Additional file 2 Characteristics of the 30 studied counties** [30]

| County | Cases | Annualized average incidences (/100,000) | Mean temperature⋆  (°C) | Rain⋆⋆ (mm) | Relative humidity⋆ (%) |
| --- | --- | --- | --- | --- | --- |
| Ruili | 3,442 | 348.204 | 21.2 (17.7, 24.6) | 26.53 (0, 43.03) | 73.0 (67, 80) |
| Tengchong | 9,255 | 246.049 | 20.4 (16, 24.5) | 17.80 (1, 24.7) | 65.0 (54, 77) |
| Gongshan | 300 | 136.897 | 6.7 (1.4,12.3) | 12.29 (2, 17.63) | 69.6 (61, 80) |
| Fugong | 455 | 80.657 | 12.3 (7.1, 17.7) | 16.59 (2, 27.85) | 67.6 (58, 78) |
| Mengla | 1,203 | 79.980 | 22.0 (18.9, 25.1) | 28.02 (0, 41.83) | 80.6 (77, 85) |
| Cangyuan | 859 | 65.931 | 19.8 (16, 23.4) | 23.63 (0, 37.93) | 72.4 (65, 81) |
| Menglian | 735 | 55.274 | 20 (16.6, 23.1) | 32.56 (0, 51.6) | 75.3 (70, 82) |
| Jinping | 966 | 47.375 | 16.5 (12.7, 20.7) | 28.95 (2.25, 40.35) | 84.8 (81, 91) |
| Longyang | 1,976 | 37.041 | 16.6 (12.1, 20.7) | 17.94 (0.08, 27.23) | 73.1 (66, 81) |
| Congjiang | 688 | 34.928 | 19 (12.3, 25.7) | 22.03 (0.7, 33.8) | 78.6 (73, 84) |
| Jiangcheng | 142 | 22.283 | 19.1 (15.7, 22.4) | 41.89 (0.38, 69.28) | 79.2 (76, 84) |
| Menghai | 420 | 21.036 | 22.8 (19.8, 25.6) | 23.17 (0, 38.63) | 77.4 (72, 84) |
| Weixi | 174 | 18.725 | 7.0 (1.6, 12.8) | 11.76 (0, 17.93) | 65.9 (58, 74) |
| Shuangjiang | 113 | 10.580 | 18.3 (14.6, 21.6) | 21.22 (0.08, 32.63) | 67.6 (59, 77) |
| Simao | 119 | 8.132 | 19.3 (16.2, 22.3) | 27.18 (0, 43.8) | 75.9 (71, 83) |
| Mojiang | 173 | 7.565 | 24.1 (20.1, 28.1) | 15.40 (0, 21.4) | 66.6 (59, 75) |
| Jingdong | 166 | 7.382 | 19 (14.6, 23.1) | 22.21 (0.38, 31.23) | 74.7 (70, 82) |
| Dechang | 86 | 7.335 | 17.6 (13.1, 22.2) | 18.39 (0, 28.1) | 59.3 (50, 71) |
| Gejiu | 156 | 5.535 | 19.5 (16.1, 23.2) | 15.90 (0, 21.18) | 68.3 (63, 75) |
| Dushan | 102 | 4.984 | 15.6 (9.7, 22) | 23.94 (1.5, 32.68) | 79.4 (73, 88) |
| Changshun | 53 | 3.598 | 16.4 (10.3, 22.6) | 22.30 (1.48, 29.1) | 77.5 (72, 84) |
| Liping | 75 | 2.522 | 16.3 (9.2, 23.8) | 23.68 (1.9, 33.33) | 81.3 (74, 90) |
| Wenshan | 64 | 2.325 | 16.5 (12.9, 20.6) | 17.64 (0.5, 26.03) | 78.3 (74, 85) |
| Wangmo | 29 | 1.609 | 20.0 (14.8, 25.6) | 22.43 (0.5, 26.43) | 73.2 (67, 80) |
| Guangnan | 74 | 1.575 | 17.5 (13.1, 22.4) | 16.98 (0.5, 23.8) | 76.8 (72, 84) |
| Weishan | 28 | 1.482 | 15.5 (11.5, 19.5) | 20.37 (0, 33.28) | 66.2 (55, 78) |
| Nanhua | 19 | 1.318 | 16.5 (12.3, 20.5) | 15.66 (0, 24.08) | 68.2 (59, 80) |
| Weng’an | 32 | 1.263 | 15.9 (9.1, 22.8) | 19.65 (2.6, 27.85) | 77.1 (71, 85) |
| Eshan | 11 | 1.156 | 16.4 (12.3, 20.3) | 16.23 (0, 23.63) | 72.6 (67, 81) |
| Huili | 29 | 1.089 | 15.6 (10.7, 20.3) | 21.53 (0, 27.95) | 68.0 (60, 77) |

⋆: weekly mean, and the two values in the parenthesis are 25 and 75% percentiles, respectively.

⋆⋆: weekly total, and the two values in the parenthesis are 25 and 75% percentiles, respectively.
